# Morphological Identification and Development of Neurite in *Drosophila* Ventral Nerve Cord Neuropil

**DOI:** 10.1371/journal.pone.0105497

**Published:** 2014-08-28

**Authors:** Guangming Gan, Huihui Lv, Wei Xie

**Affiliations:** 1 The Key Laboratory of Development Genes and Human Diseases, Ministry of Education, Institute of Life Sciences, Southeast University, Nanjing, China; 2 School of Medicine, Southeast University, Nanjing, China; Rutgers University, United States of America

## Abstract

In *Drosophila*, ventral nerve cord (VNC) occupies most of the larval central nervous system (CNS). However, there is little literature elaborating upon the specific types and growth of neurites as defined by their structural appearance in *Drosophila* larval VNC neuropil. Here we report the ultrastructural development of different types VNC neurites in ten selected time points in embryonic and larval stages utilizing transmission electron microscopy. There are four types of axonal neurites as classified by the type of vesicular content: clear vesicle (CV) neurites have clear vesicles and some T-bar structures; Dense-core vesicle (DV) neurites have dense-core vesicles and without T-bar structures; Mixed vesicle (MV) neurites have mixed vesicles and some T-bar structures; Large vesicle (LV) neurites are dominated by large, translucent spherical vesicles but rarely display T-bar structures. We found dramatic remodeling in CV neurites which can be divided into five developmental phases. The neurite is vacuolated in primary (P) phase, they have mitochondria, microtubules or big dark vesicles in the second (S) phase, and they contain immature synaptic features in the third (T) phase. The subsequent bifurcate (B) phase appears to undergo major remodeling with the appearance of the bifurcation or dendritic growth. In the final mature (M) phase, high density of commensurate synaptic vesicles are distributed around T-bar structures. There are four kinds of morphological elaboration of the CV_I_ neurite sub-types. First, new neurite produces at the end of axon. Second, new neurite bubbles along the axon. Third, the preexisting neurite buds and develops into several neurites. The last, the bundled axons form irregularly shape neurites. Most CV_I_ neurites in M phase have about 1.5–3 µm diameter, they could be suitable to analyze their morphology and subcellular localization of specific proteins by light microscopy, and they could serve as a potential model in CNS *in vivo* development.

## Introduction

Neurites are swollen projections that form from neuronal somata, dendrites or axons. Axonal neurites are often characterized with synapses, synaptic vesicles, microtubules, and mitochondria in TEM. Synapses, located in the free surface of an axonal neurite, are communication connections between axonal neurites and target cells, the main site of nerve signal processing and neural information transmission, and a bridge the neural network circuit. Neurites undergo several dynamic processes, such as neurite growth, extension, retraction, and branching, which are regulated by many molecules [1]–[3].

There are, however, notable differences between mammalian and insect neurite morphology. In the *Drosophila* NMJ, type I boutons are repeatedly wrapped by the subsynaptic reticulum (SSR) that is formed by the muscle cell membrane [4], [5], a structure that does not exist in mammalian neurites. In the insect CNS, several dendrites gather opposite one pre-synapse structure and form multiple postsynaptic sites [6]–[9] which serve to increase the efficiency of the nerve signal transformation. However, in mammals there is only a single postsynaptic site [10]. During insect synaptic formation, synaptic vesicles dock and calcium channels gather in unique structures known as T-bar structures [11], and the Bruchpilot (Brp) protein involves in the assembly [12], [13].

Depending on different classification criteria, the NMJ boutons and brain boutons can be divided into different types in *Drosophila*. Adult *Drosophila* NMJs are classified by two distinct types of synaptic boutons (types I and II). Type II boutons are small (0.5–1.5 µm in diameter) and contain octopamine. Type I boutons are densely packed with clear synaptic vesicles, are comparatively larger (the diameters 0.8–5.5 µm), and contain glutamate [14]. Larval *Drosophila* NMJs are classified by three types synaptic boutons (types I, II, and III) according to the size, characteristics of SSR, and compositions of synaptic vesicles. Larval type I boutons are divided into type-Ib and type-Is. The type-Ib boutons, characterized by a thick SSR, are the largest, with diameters of 3–5 µm [4], and contain clear synaptic vesicles that carry glutamate [5]. The type-Is boutons, characterized by a less developed SSR, are smaller, with diameters of 1–1.5 µm [4], and contain clear- and dense-core vesicles [5]. Both type-II and type-III terminals lack the distinctive SSR found in type I. Type II boutons are smaller than 2 µm and contain both dense-core vesicles and small clear vesicles, which carry glutamate and octopamine, respectively [15]. Type III boutons have intermediate diameters and contain mainly dense-core vesicles of different sizes and densities [5], which carry glutamate, insulin-like peptide [16], and leucokinin-1 [17].

In adult *Drosophila* mushroom body calyx, there are three morphological types of PN boutons according to the synaptic vesicle composition (CCV-PNs, DCVPNs and DB-PNs). CCV-PNs (clear-core vesicle-projection neurons) have exclusively clear-core synaptic vesicles, while DCVPNs (dense-core vesicle-projection neurons) have mixed clear- and dense-core vesicles. DB-PNs (dark bouton-projection neurons) have a dark cytoplasm, with both clear- and dense-core vesicles [18]. There are four classes of neurite in the *Drosophila* first instar larval neuropils of brain and VNC: they are termed globular, varicose, axiform, and dendritiform, according to characteristics of their appearance and synapse (big neurites can be called boutons) [19]. Globular and varicose neurites have large diameter segments that carry almost exclusively presynaptic sites, while dendritiform neurites and axiform neurites are thin [19]. VNC accounts for the considerable proportion of the nerve tissue in the adult *Drosophila* [20], which suggests that the *Drosophila* VNC plays an important physiological role.

With *Drosophila* development, the number of boutons increases 10-fold [4], [21], and the new NMJ boutons form by “bud”, “divide”, or “de novo” [22].

However, little literature has reported the fine ultrastructure and development of the complex, seemingly disorderly neurites in *Drosophila* CNS. Here, we analyzed the morphological classification and growth of *Drosophila* VNC axon neurite in ten periods from embryo to larvae, and identified four types of axon neurites, five developmental phases of the CV neurite, four kinds of growth manners of CV_I_ neurites in TEM.

## Results

### 1. Development of *Drosophila* larval VNC and the ultrastructure of neuropil

**Figure 1:**
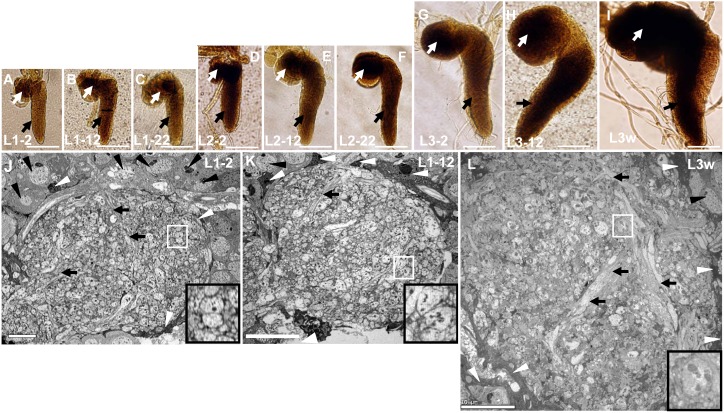
Development and ultrastructure of *Drosophila* larval VNC. The development process of *Drosophila* larval VNC (**A–I**). VNC appearance and length are not significantly different in the periods of L1–2 (**A**), L1–12 (**B**), L1–22 (**C**) and L2–2 (**D**). The length of VNC in the L2–12 period (**E**) was longer than the L2–2 period (**D**), and once again with no obvious difference between L2–12 period and L2–22 period (**F**). The shape and the length of VNC display an obvious change during the L3–2 (**G**), L3–12 (**H**) and L3w (**I**) periods. White arrow indicates the brain, and black arrow indicates VNC. The ultrastructure of neuropils at low magnification in L1–2 period (**J**), L1–12 period (**K**) and L3w period (**L**). The VNC neuropil is wrapped by inner glial cells, and they were filled with nerve fibers and neurites (**J**–**L**). The size of neurites and neuropil in L1–2 period (**J**) are not obviously different than the L1–12 period (**K**), but size of neurites and neuropil in the L3w period (**K**) is bigger than L1–2 period (**J**) and L1–12 period (**K**). Most of the neurites are in S phase and T phase in L1–2 period (**J**) and L1–12 period (**K**). The black triangle indicates the neuronal soma, the white triangle indicates the inner glial cell and the thick arrow indicates nerve fiber. The large black box is enlarged from the small white box in the same diagram, and indicates the CV_I_ neurite. Scale Bar A–I: 50 µm; J–K: 5 µm; L: 10 µm.

In *Drosophila*, VNC occupied most of CNS in larvae stages (Fig. 1A–I). VNC developed slowly in *Drosophila* first instar larvae. There was no obvious difference in VNC appearance and length in the L1–2 (Fig. 1A), L1–12 (Fig. 1B), and L1–22 period (Fig. 1C). Even the VNC appearance and length in L2–2 period (Fig. 1D) showed no obvious differences when compared with the L1–2, L1–12, and L1–22 period. VNC developed quickly in the early stage of *Drosophila* second instar larvae. VNCs in L2–12 period (Fig. 1E) were longer than those in the L2–2 period (Fig. 1D), but VNC length was not obviously different between the L2–22 period (Fig. 1F) and L2–12 period (Fig. 1E). The appearance and length of VNCs displayed obvious changes in L3–2 (Fig. 1G), L3–12 (Fig. 1H) and L3w period (Fig. 1I), which suggests that VNC developed quickly in entire third instar larvae. Thus the development of *Drosophila* larval VNC was a non-linear process.

We analyzed the ultrastructures of neuropils in 10 developmental periods in *Drosophila* VNCs. The *Drosophila* VNC consists of cortex and neuropil, with the cortex consisting primarily of neurons (Fig. 1J–L) and glial cells. The larvae neuropils in VNCs were wrapped by inner glial cells, but no glial cells were present inside of the neuropils (Fig. 1J–L). The larvae neuropils primarily consisted of nerve fibers and different types of neurites (Fig. 1J–L) which occupied most regions of the neuropil according to the ultrastructures in 10 developmental periods.

### 2. Identification of four classes of axon neurites in *Drosophila* VNC neuropil

Neurites were divided into dendrite neurites and axon neurites in *Drosophila* VNC neuropil. Axon neurite were further subdivided into four types: clear vesicles (CV) neurite, dense-core vesicles (DV) neurite, mixed vesicles (MV) neurite and large vesicles (LV) neurite, according to the size and inclusions of vesicles.

**Figure 2:**
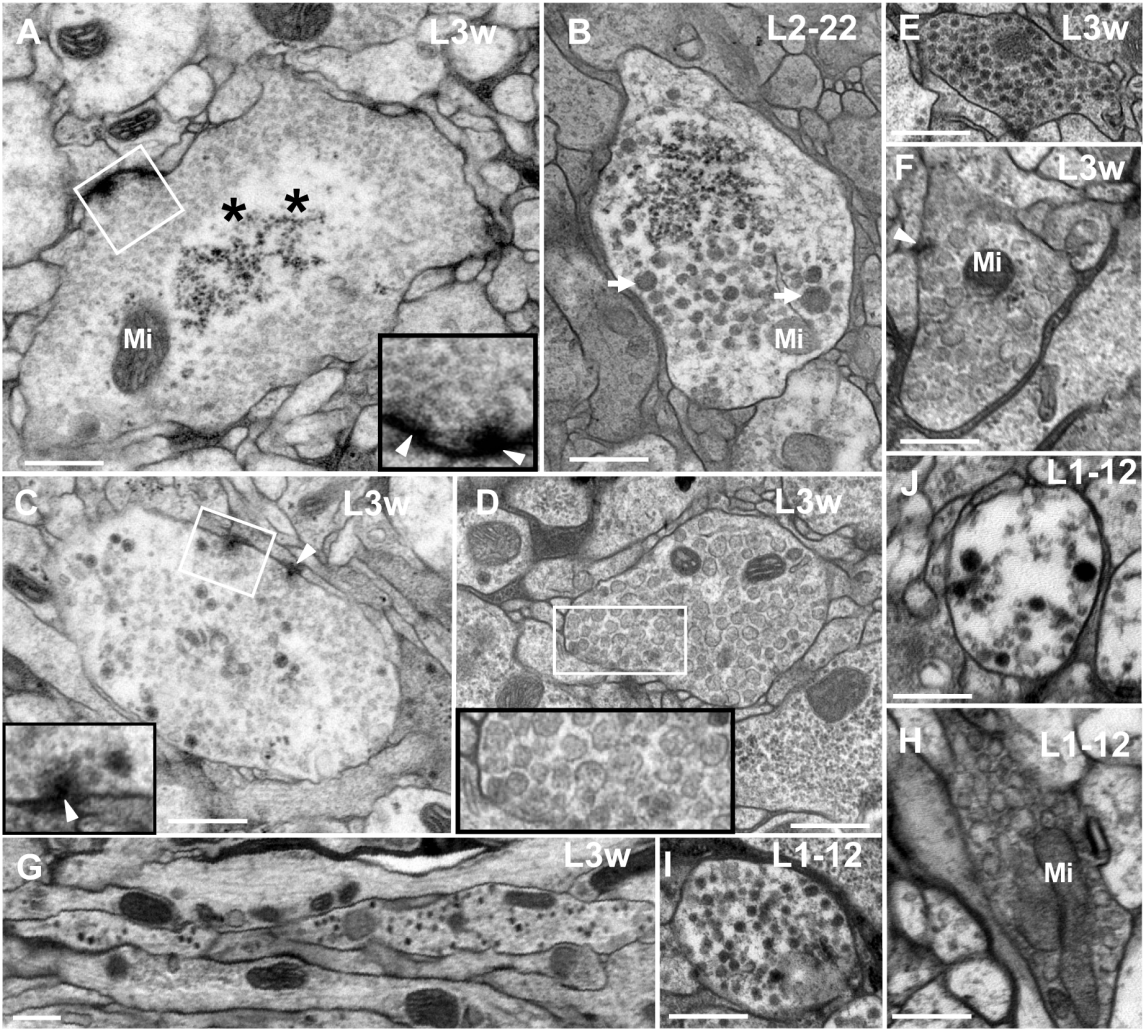
Identification four classes of neurites in *Drosophila* VNC. Clear vesicle (CV) neurite contains clear vesicles at L3w period and the CV_I_ is light-colored and globular (**A**); Dense-core vesicle (DV) neurite contains dark dense-core vesicles (**B**, **E** and **I**). DV_I_ neurite contains the similar size of dark dense-core vesicles (**E**, **I**), and DV_II_ neurite contains bigger and different size dark dense-core vesicles (**B**). Mixed vesicle (MV) neurite contains both clear and dense-core vesicles (**C**, **J**). Large vesicle (LV) neurite contains large and spherical translucent vesicles (**D**, **F** and **H**). All the DV neurites, MV neurites and LV neurites are globular (**A**–**I**). In the same diagram, the large black box is enlarged from the small white box. **G** indicates the DV vesicles in an axon at L3w period. Triangle indicates synapse, arrow indicates big size dense-core vesicle, asterisk indicates the central region of CV neurite and Mi indicates mitochondria. Scale Bar: 500 nm.

**Figure 3:**
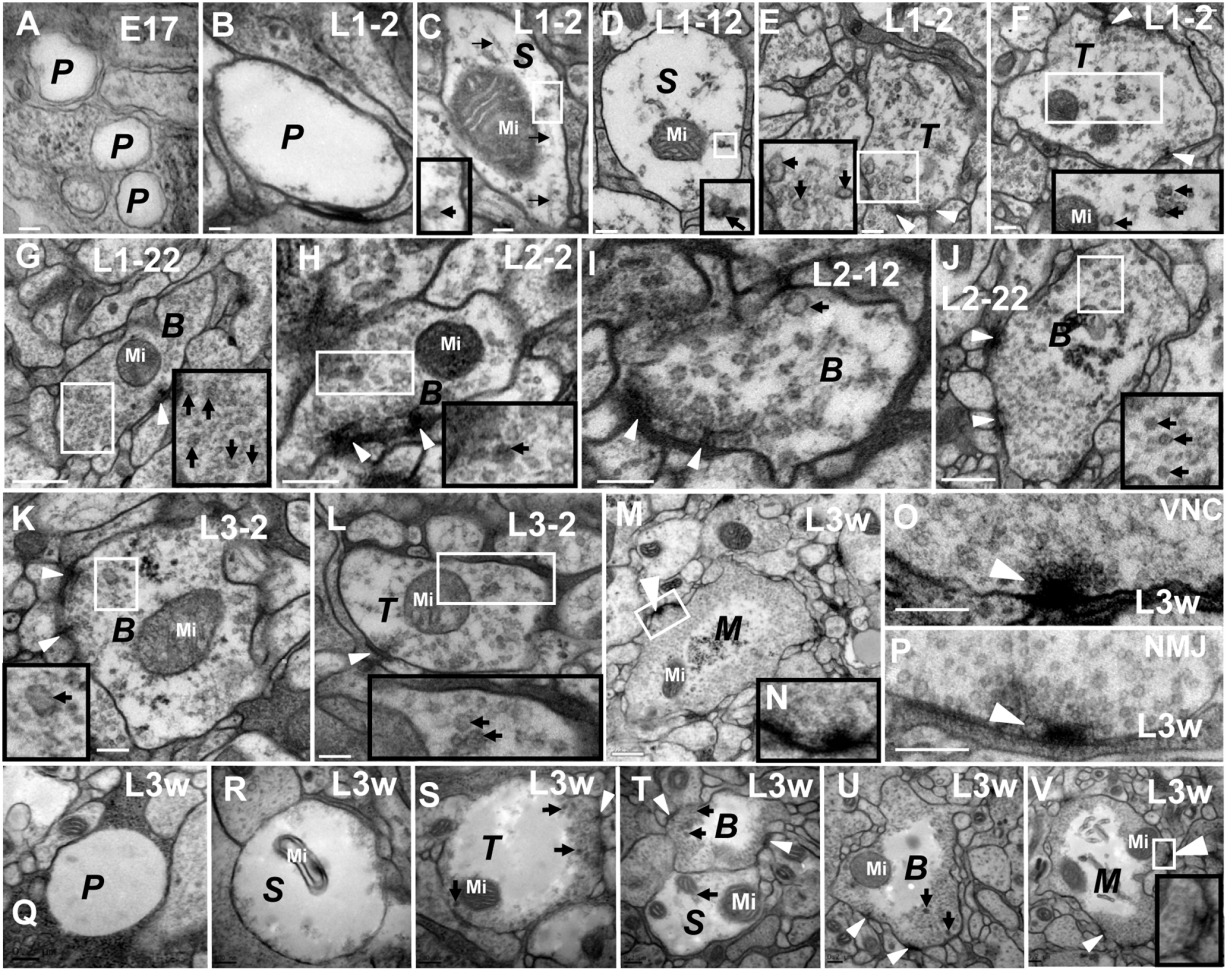
The CV_I_ neurites are divided into five developmental phases. In P phase (**A**–**B**, **Q**), there are no synapses, microtubules, or synaptic vesicles in the neurite. In S phase (**C**–**D**, **R** and **T**), there are some microtubules, mitochondria, or a few small or big dark synaptic vesicles, but no synapse in the neurite. In T phase (**E**–**F**, **L** and **S**), the synapses have immature T-bar structures, a few synaptic vesicles gathered around the small T-Bar structure, and microtubules disappeared in the neurite. In B phase (**G**–**K**, **T**–**U**), the neurites look bifurcated or dendritic, the T-bar structure is incomplete, and the two different diameters of synaptic vesicles are still present. In M phase, (**M**–**O**, **V**) there are a lot of commensurate synaptic vesicles cluster around the typical T-bar structure, and few big dark synaptic vesicles; **P** shows the NMJ T-bar structure. All the neurites in P phase (**Q**), S phase (**R**, **T**), T phase (**S**), B phase (**T**–**U**) and M phase (**V**) can be observed simultaneously at L3w period, and there are no synaptic vesicles in the center of the big neurites (**Q**–**V**). In the same diagram, the large black box is enlarged from the small white box, and **N** is the magnification of the **M** diagram box. ***P*** indicates P phase, ***S*** indicates S phase, ***T*** indicates T phase, ***B*** indicates B phase and ***M*** indicates M phase. Triangle indicates synapse, thick arrow indicates the big dark synaptic vesicle and thin arrow indicates microtubule (B–D), and Mi indicates mitochondria. Scale Bar A–C: 100 nm; D–F, H–I, K–L, O–P: 200 nm; G, J, M: 500 nm.

**Figure 4:**
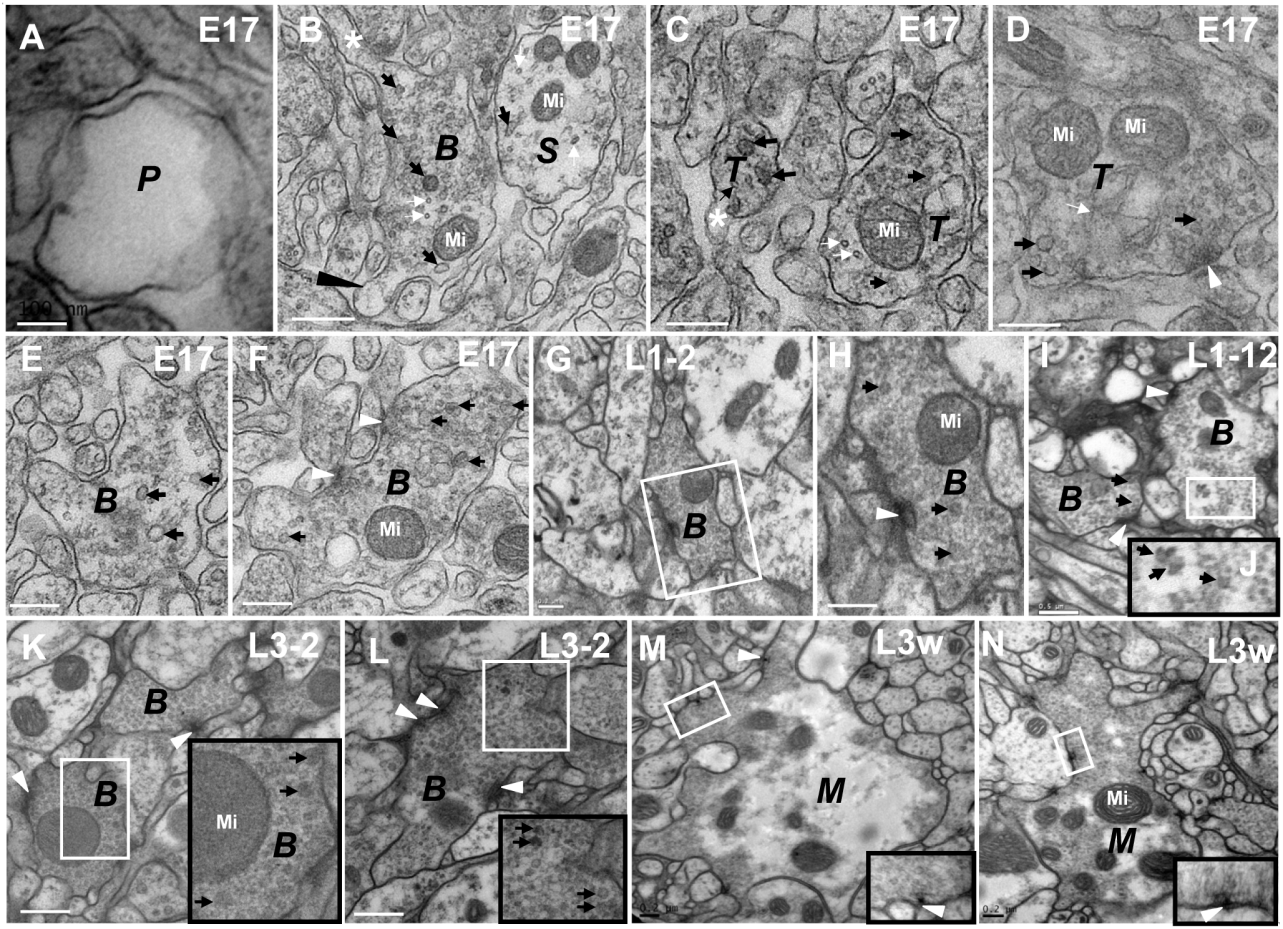
The CV_II_ neurites are divided into five developmental phases. In P phase (**A**), there are not synapses, microtubules, or synaptic vesicles in the neurite. In S phase (**B**), there are some microtubules, mitochondria, or different sizes of synaptic vesicles, but no synapses. In T phase (**C**–**D**), there are residual microtubules and a greater variety in the size of synaptic vesicles in the neurite; the synapse is immature, and there is no T-bar structure formation. In B phase (**B**, **E**–**L**), the neurites look bifurcated or dendritic, and the two different diameters of synaptic vesicles remain present. In M phase, there are dark cytoplasm, typical T-bar structures and a lot of commensurate synaptic vesicles cluster around it, but the appearance of the neurite remain bifurcated or dendritic, and have several branches around it (**M**–**N**). In embryo stage, the synapses have not T-bar structures (**F**), but have the immature T-bar structures and dark cytoplasm throughout the larval stage (**G**–**L**) in B phase. In the same diagram, the large black box is enlarged from the small white box, and **H** is the magnification of the **G** diagram box. Triangle indicates synapse, thick arrow indicates the big dark synaptic vesicles, thin arrow indicates microtubules, asterisk indicates the axon and black long triangle indicates the nascent branch of neurite in B phase (**B**). ***P*** indicates P phase, ***S*** indicates S phase, ***T*** indicates T phase, ***B*** indicates B phase, ***M*** indicates M phase and Mi indicates mitochondria. Scale Bar A: 100 nm; B–H, K–M: 200 nm; I: 500 nm.

**Figure 5:**
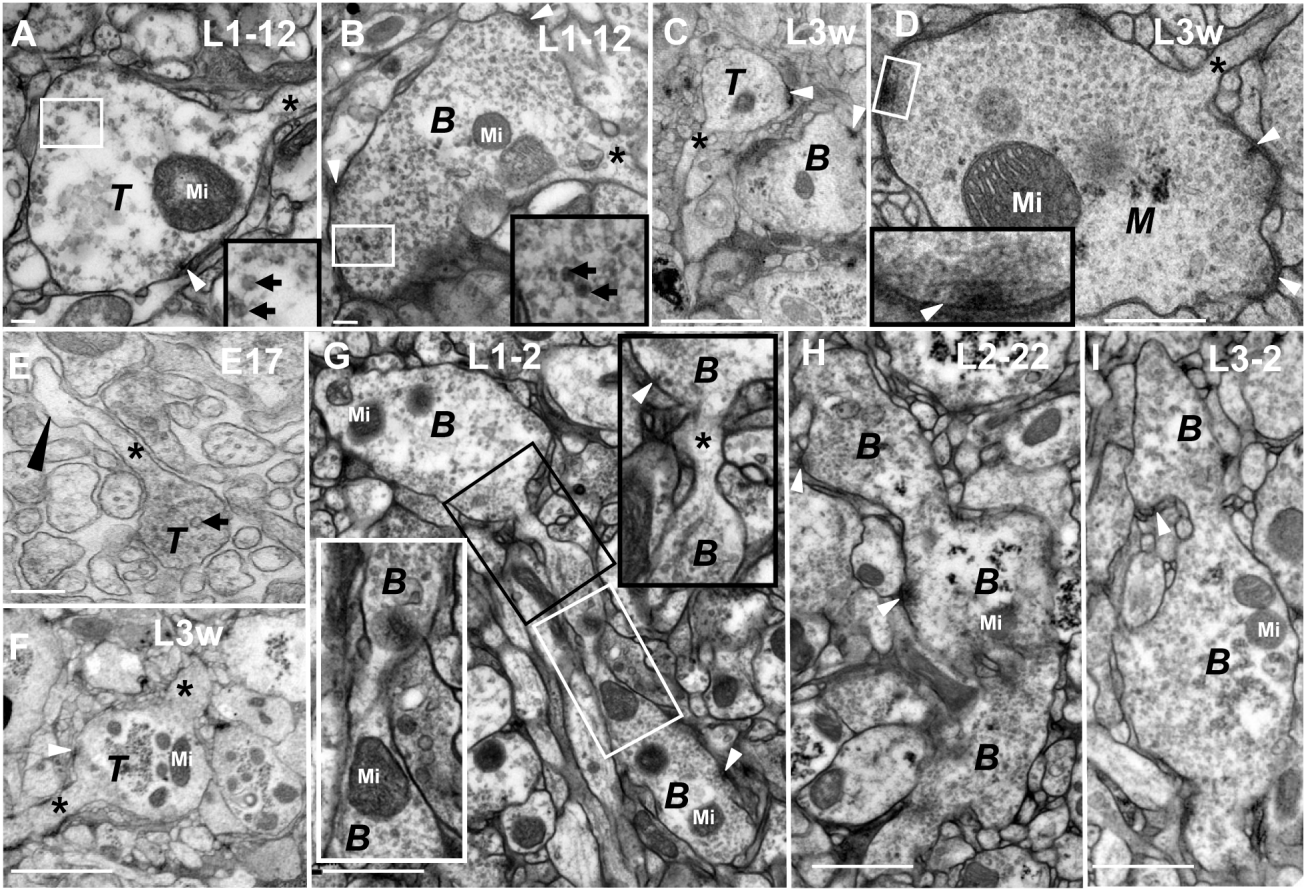
The new neurites produce via budding at the end of axon and bubbling along the axon. **A**–**E** shows the new neurites bud at the end of axons in different phases, and the terminal of the fine axon is connected to intumescent neurite. **A**, **C** and **E** show the neurites in T phase at early stage 17 embryo (**F**), L1–2 period (A) and L3w period (**C**); **B**–**C** show the neurites in B phase in L1–12 period (**B**) and L3w period (**C**); **D** shows the neurite in M phase in L3w period. **E**–**I** shows neurites bubbling along the axons, and three neurites in B phase grow on one axon in L1–2 phase (G) and L2–22 phase (**H**). **I** diagram shows two neurites in B phase bubbling along the axon in L3–2 period. **E** shows the nascent neurite in P phase bubbling along the axon. **F** shows two axons beside one neurite in T phase. In **G** diagram, the large black box is enlarged from the small white box, and the large black box is enlarged from the small white box. ***T*** indicates T phase, ***B*** indicates B phase and ***M*** indicates M phase. White triangle indicates synapse, black long triangle indicates the neurite in P phase, arrow indicates the big dark synaptic vesicles, asterisk indicates the axon and Mi indicates mitochondria. Scale Bar A–B, E: 200 nm; C, F: 2000 nm; D: 500 nm; G–I: 1000 nm.

CV neurites contained large numbers of clear vesicles that were distributed around the neurite, few synaptic vesicles in the center of the CV neurite, and several T-bar structures in L3w period (Fig. 2A; Fig. 3V; Fig. 5D). The ultrastructure of the T-bar in CV neurite (Fig. 3O) looked very similar to the NMJ bouton (Fig. 3P)[5], [6]; it was composed of an electron-dense stalk and a bar perpendicular to the stalk surrounded by abundant of synaptic vesicles (Fig. 2A; Fig. 3O; Fig. 4M–N; Fig. 5D). The CV neurites were divided into two subtypes, CV_I_ and CV_II,_ according to color and appearance of neurite. CV_I_ neurites were numerous and widely distributed throughout the neuropil. They were light-colored and had synaptic vesicles with a diameter of 32.87 nm±3.9 (n = 277, 8 neurites; Fig. 8A), and most of the CV_I_ neurites were globular (Fig. 1L; Fig. 2A; Fig. 3V; Fig. 5D) in L3w period. However, the CV_II_ neurites had dark cytoplasm, looked bifurcated or dendritic, and were rare throughout the whole larvae stage (Fig. 4G–N). Furthermore, there was a greater variation in appearance of both the CV_I_ neurites (Fig. 3; Fig. 9) and CV _II_ neurites (Fig. 4; Fig. 10) with the development of VNC.

DV neurites contained dark dense-core vesicles that almost filled the whole neurite (Fig. 2B, E, I). They were globular during all the developmental periods, distributed throughout the peripheral layer of neuropil, and were infrequently encountered. We observed more than 50 DV neurites in which no T-bar structures were found. DV neurites could be divided into DV_I_ (Fig. 2E, I) and DV_II_ (Fig. 2B) according to vesicle size. DV_I_ neurites had smaller uniform vesicles measuring 63.74 nm±9.4 (N = 81, 4 Neurites; maximum diameter is 87.4 nm), but DV_II_ neurite vesicles were bigger, measuring 97.29 nm±24.0 (N = 61, 5 neurites) in L3w period (Fig. 8A), with some dense-core vesicles measuring more than 179.1 nm in the DV_II_ neurites. DV neurites formed in L1–2 period, and the DV_I_ neurites were found at L1–12 period (Fig. 2J). DV vesicles could be observed in the axon until L3w period (Fig. 2G).

MV neurites contained both clear and dense-core vesicles that nearly filled the whole neurite (Fig. 2C, J). They were globular and were sparsely scattered throughout the peripheral layer of neuropil. In MV neurites, there were several T-bar structures around which both the clear and dense-core vesicles gathered (Fig. 2C). The clear vesicles were similar in appearance and size (32.2 nm±3.3, N = 69, 7 neurites) to the clear vesicles observed in CV_I_ neurites (Fig. 8A). Furthermore, the dense-core vesicles (64.35 nm±7.4, N = 69, 7 neurites) were similar to dense-core vesicles in DV_I_ neurites in both appearance and size (Fig. 8A). MV neurites formed during the L1–2 period, and we could observe the dense-core vesicles and clear vesicles in MV at L1–12 period (Fig. 2J).

LV neurites were a newly discovered structure and were not reported in the *Drosophila* bouton or neurite. They are filled with large and spherical translucent vesicles that had a diameter of 129.5 nm±26.3 (N = 125, 11 neurites; Fig. 8A) in L3w period (Fig. 2D, F), and some large vesicles were measured more than 209.6 nm in the LV neurites. T-bar structures were very rare in the LV neurite (Fig. 2F White triangle). In more than 30 observed LV neurites, only one T-bar structure was found in the L3w period. The LV neurite was characterized by a globular shape and was sparsely distributed in the peripheral layer of neuropil. It could also be observed in the neuropil of adult wild-type *Drosophila* brain (data not shown). LV neurites formed in L1–2 period, and we could observe the huge vesicles characteristic of LV neurites during the L1–12 period (Fig. 2H).

There were no statistically significant differences between clear vesicles in CV neurites and MV neurites, and between dense-core vesicles in DV_I_ neurites and MV neurites. However, there were extremely statistically significant differences between dense-core vesicles in DV_I_ neurites and DV_II_ neurites, between clear vesicles in CV neurites and dense-core vesicles in DV_I_ or DV_II_ neurites clear vesicles, and between vesicles in DV_II_ neurites and LV neurites (Fig. 8A).

### 3. *Drosophila* CV neurite morphogenesis from P phase to M phase

All the DV neurites (Fig. 2B, E, I), MV neurites (Fig. 2C, J), and LV neurites (Fig. 2F, H) were globular in appearance and the vesicles of these three types of neurites did not display obvious change throughout the entire larval stage. However, the CV neurites (both CV_I_ and CV_II_) dramatically changed in size (Fig. 1J–L) and other features. We identified five developmental phases according to appearance and cell contents (i.e. synaptic vesicles, synapses, microtubules, and mitochondria) during CV neurite morphogenesis: the primary (P) phase, the second (S) phase, the third (T) phase, the bifurcate (B) phase, and the mature (M) phase. There were similar developmental processes between CV_1_ neurites and CV_II_ neurites.

P phase was the first stage of CV neurite development. In P phase, the CV neurite contained vacuolated projection in which there was no synapse, synaptic vesicle, microtubule, or mitochondrion, both in the CV_I_ neurites (Fig. 3A–B, Q; Fig. 5E) and CV_II_ neurites (Fig. 4A). We observed the CV_I_ neurites in P phase originated in early stage 17 embryos (Fig. 3A; Fig. 5E) and the entire larval stage, including L3w period (Fig. 3Q). However CV_II_ neurites emerged only in early stage 17 embryos (Fig. 4A).

**Figure 6:**
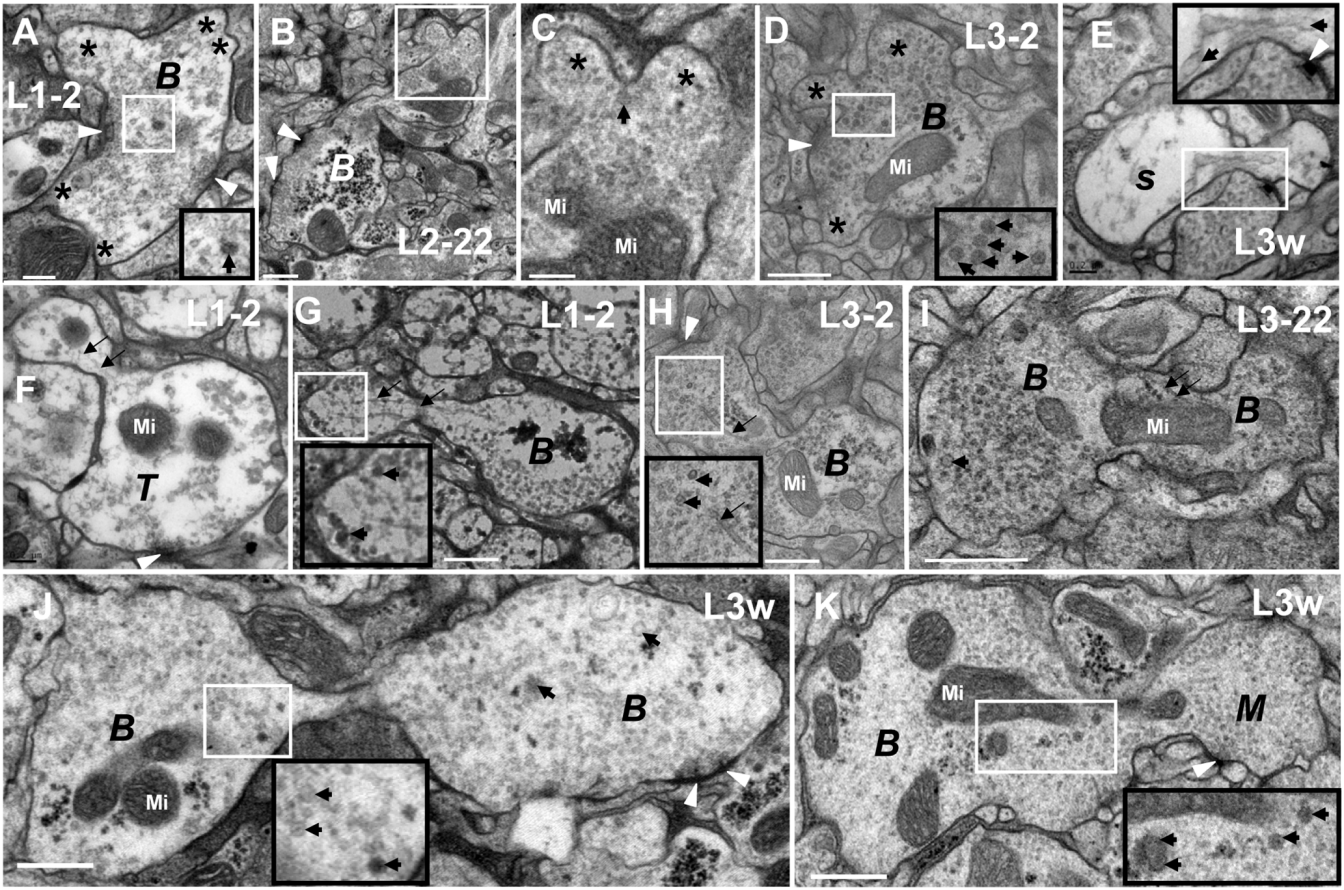
The preexisting neurite buds, divides and produces the CV_I_ neurites. **A** shows the neurite in B phase issue five branches (asterisk indicates) at L1–2 period, and two of the branches (upper right corner) have no synaptic vesicle. **B**–**C** shows the neurite issues two branches in B phase at L2–22 period. **D** shows the neurite issues three branches in B phase at L3–2 period. **E**–**K** shows the preexisting neurite divides and produces a new neurites. **F**–**I** shows the big furrow and some microtubules between the preexisting neurite and the new neurite. J shows the furrow is narrow, with no microtubules visible in it. The right part of **K** shows the new neurite developing into M phase, and the uniform distribution of synaptic vesicles around the neurite. **E** shows the neurite in S phase divides and produces the new neurite at L3w period. In the same diagram, the large black box is enlarged from the small white box. Triangle indicates synapse, thick arrow indicates the big dark synaptic vesicle, thin arrow indicates microtubule, asterisk indicates the branch of B neurite and Mi indicates mitochondria. ***S*** indicates S phase, ***T*** indicates T phase, ***B*** indicates B phase and ***M*** indicates M phase. Scale Bar A, E–F: 200 nm; B–D, G–K: 500 nm.

**Figure 7:**
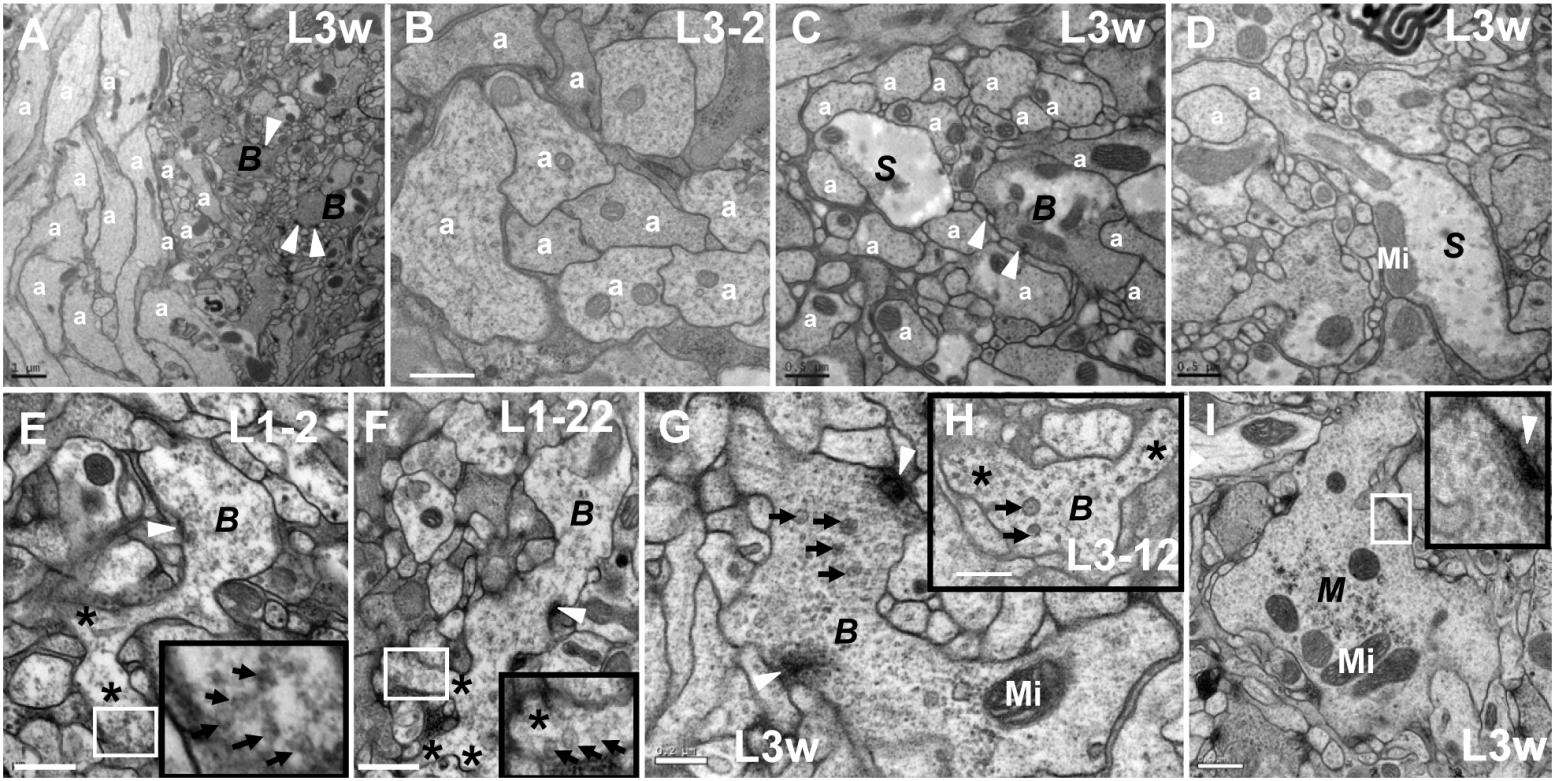
Main axons expand and form irregularly CV_I_ neurites. **A** shows the longitudinal section of a nerve fiber that is made up of the main axons whose diameter is roughly similar to some neurites in B phase; **B** shows the cross-section of a nerve fiber that is made up of main axons; **C** shows one of the main axons is developing into S phase, and another is developing into B phase in the cross-section. **D** shows one of main axons is developing into S phase in the longitudinal section. The main axon forms into irregularly shaped neurites in B phase and three branches in L1–2 period (**E**) and L1–22 period (**F**), with some big dark synaptic vesicles. **H** shows the main axon forms an irregularly shaped neurite and two branches in L3–12 period, with some big dark synaptic vesicles. **G** shows the main axon forms irregularly shaped neurites in L3w period, with some big dark synaptic vesicles and typical T-bar structures. **I** diagram shows another main axon forms irregularly shaped neurites in L3w period, with some typical T-bar structures but few big dark synaptic vesicles. a indicates axon, ***S*** indicates S phase, ***B*** indicates B phase and ***M*** indicates M phase. Arrow indicates the big dark synaptic vesicles, triangle indicates synapse, asterisk indicates the branch and Mi indicates mitochondria. In the same diagram, the large black box is enlarged from the small white box. Scale Bar A: 1000 nm, B–F, H–I: 500 nm; G: 200 nm.

**Figure 8:**
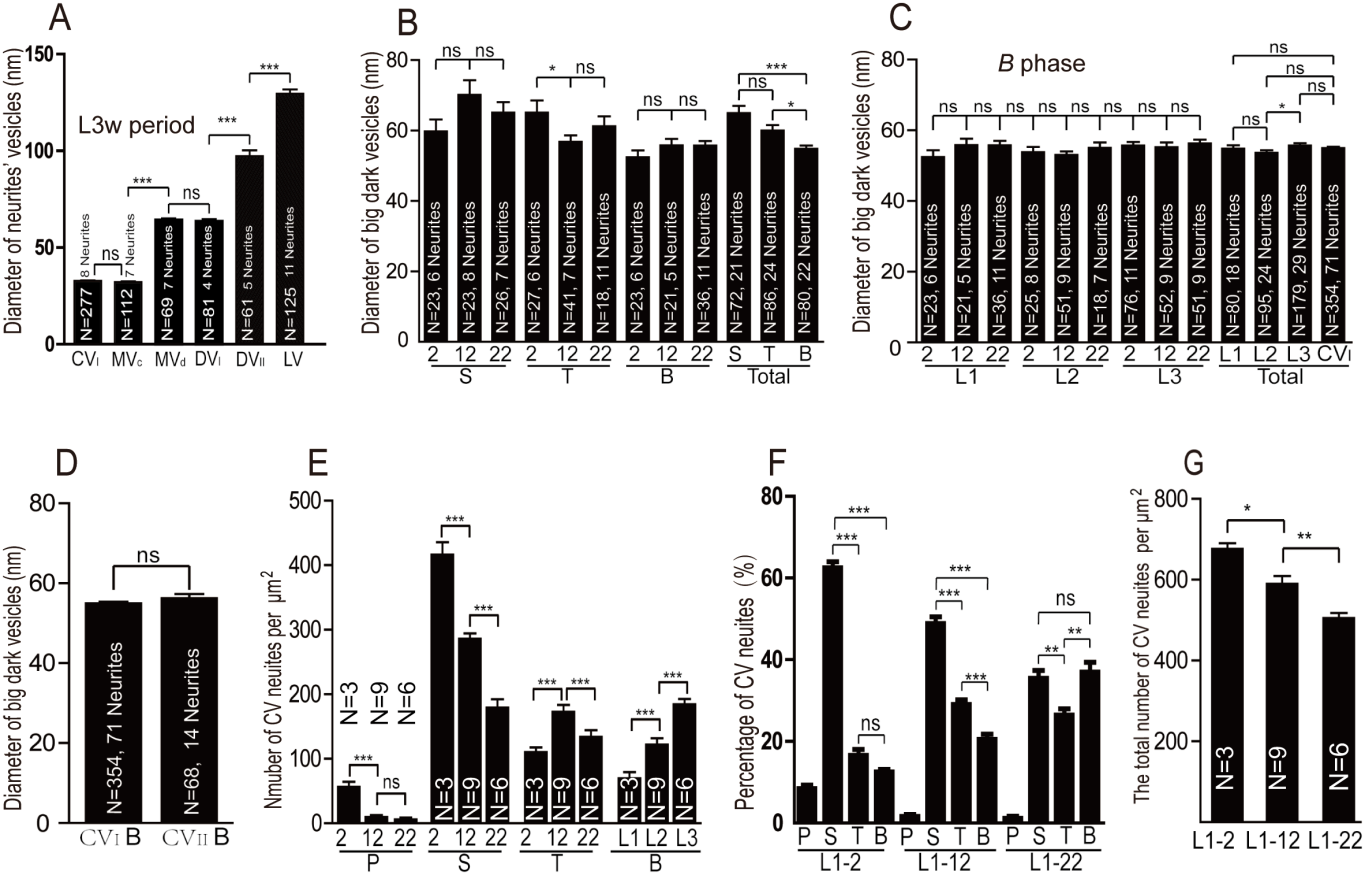
Characterization of vesicles and neurites in different developmental periods. **A–D** shows the quantifications of vesicles’ diameters of in neurites. **A** shows the size of vesicles in four types of neurites at L3w period. **B** shows the diameter of big dark synaptic vesicles in developmental CV_I_ neurites in the first instar larvae. The big dark synaptic vesicles in T phase are smaller in L1–12 period when compared to L1–2 period (P = 0.035). **C** shows the big dark synaptic vesicles do not show statistically significant difference at developmental periods, and among the first instar larval stage, the second instar larval stage and third instar larval stage, however, there is statistically significant difference between the first instar larval stage and the second instar larval stage (P = 0.043). **D** shows no difference of total big dark synaptic vesicles between CV_I_ and CV_II_ neurites in the whole instar larval stage. **E–G** shows the quantifications of the density and percentage in the first instar larval CV neurites. **E** shows the CV neurites decreases in P and S phase, but increases in B phase from L1–2 period to L1–22 period. However, the CV neurites, in T phase, increases significantly from L1–2 period to L1–12 period, but decreases from L1–12 period to L1–22 period. **F** shows percentage of CV neurites in P, S, T, B phase. From L1–2 period to L1–12 period, the percentage of S phase is the highest. At L1–22 period, the percentage of S and B phase are the highest. **G** shows the number of total CV neurites including P, S, T and B phase decreases from L1–2 period to L1–12 period, and from L1–12 period to L1–22 period. **S** indicates S phase, **T** indicates S phase, **B** indicates B phase. N = Number of samples. Error bar indicates SEM, t test. *p<0.05, **p<0.01, ***p<0.001, ns = non-significant.

S phase was the second stage of CV neurite development. In S phase, there were some inclusions such as microtubules (Fig. 3C; Fig. 4B), mitochondria (Fig. 3C–D, R, T; Fig. 4B; Fig. 7C–D), or a few small synaptic vesicles and big dark synaptic vesicles (Fig. 3D; Fig. 4B; Fig. 6E), but no synapse in either CV_I_ neurites or CV_II_ neurites. In S phase, the big dark synaptic vesicles of CV_I_ neurites was 59.8 nm±16.2 at L1–2 period (N = 23, 6 neurites; maximum diameter is 124.6 nm), 70.2±19.9 at L1–12 period (N = 23, 8 neurites; maximum diameter is 119.4 nm), and 65.1±15.5 at L1–22 period (N = 26, 7 neurites; maximum diameter is 117.2 nm). However, there were no statistically significant differences among the big dark synaptic vesicles in S phase (Fig. 8B).

**Figure 9:**
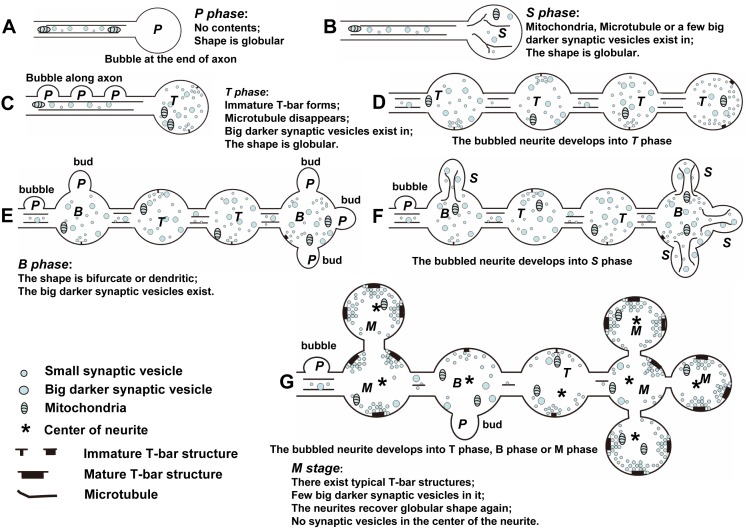
The model of CV_I_ neurite development. ***P*** indicates P phase, ***S*** indicates S phase, ***T*** indicates S phase, ***B*** indicates B phase and ***M*** indicates M phase. Note: the neurites in P phase, S phase, and T phase are globular, while the neurites in B phase are bifurcated or dendritic, and the neurites in M phase return to globular again. There are big dark synaptic vesicles in S phase, T phase, and B phase, and few big dark synaptic vesicles in M phase. The new neurites in P phase bubbled continuously along the axon throughout the larval stage.

**Figure 10:**
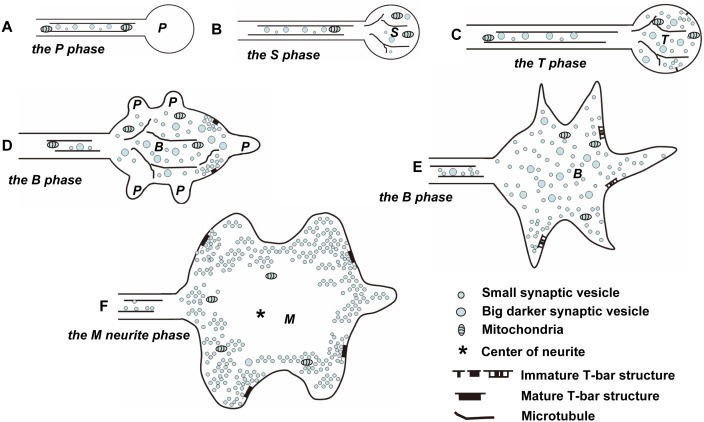
The model of CV_II_ neurite development. ***P*** indicates P phase, ***S*** indicates S phase, ***T*** indicates S phase, ***B*** indicates B phase and ***M*** indicates M phase. Note: There are microtubules in the T phase of CV_II_ neurite (C), the shape is bifurcated or dendritic in the B phase (D–E) and the M phase of CV_II_ neurite (F), but it is globular in the M phase of CV_I_ neurite.

T phase was the third stage of CV neurite development and had immature synapses in the neurites (Fig. 3E–F, L, S; Fig. 4D; Fig. 5A, C). In the larvae stage, the immature T-bar structure was small, and had no microtubules in CV_I_ neurites (Fig. 3E–F, L, S; Fig. 5A, C, F). However, the synapse had no T-bar structure (Fig. 4D), and had a few microtubules (Fig. 4C–D) in CV_II_ neurites during the early stage 17 embryos. In T phase, the small synaptic vesicles increased, and the big dark synaptic vesicles of CV_I_ neurites were 65.1 nm±17.8 at L1–2 period (N = 27, 6 neurites; maximum diameter is 123.3 nm), 56.8±11.8 at L1–12 period (N = 41, 7 neurites; maximum diameter is 95.4 nm), and 61.2±11.8 at L1–22 period (N = 18, 11 neurites; maximum diameter is 81.8 nm). However, the big dark synaptic vesicles had a statistically significant difference between the L1–2 period and L1–12 period, but no statistically significant difference between L1–12 period and L1–22 period in T phase (Fig. 8B).

B phase was the remodeling stage of CV neurite development, and both the CV_I_ neurite (Fig. 3G–K, T–U; Fig. 5B–C, G–I; Fig. 6A–D, G–J; Fig. 7A–C, E–H) and CV_II_ neurite (Fig. 4E–L) became bifurcated or dendritic, moreover the big dark synaptic vesicles always existed. However, the appearance of the neurites was globular in P phase, S phase, and T phase. The small synaptic vesicles further increased, and even completely filled the CV_II_ neurite (Fig. 4F–L).

In B phase, the big dark synaptic vesicles of CV_I_ neurites was 52.4 nm±9.2 at L1–2 period (N = 23, 6 neurites; maximum diameter 78.0 nm), 55.8 nm±8.2 at L1–12 period (N = 23, 8 neurites; maximum diameter 74.8 nm), 55.8 nm±7.6 at L1–22 period (N = 36, 11 neurites; maximum diameter 62.1 nm), 53.8 nm±7.3 at L2–2 period (N = 25, 8 neurites; maximum diameter 70.9 nm), 53.0 nm±7.3 at L2–12 period (N = 51, 9 neurites; maximum diameter 81.5 nm), 55.0 nm±6.8 at L2–22 period (N = 18, 7 neurites; maximum diameter 69.5 nm), 55.7 nm±8.4 at L3–2 period (N = 76, 11 neurites; maximum diameter 97.9 nm), 55.2 nm±9.8 at L3–12 period (N = 52, 9 neurites; maximum diameter 77.9 nm) and 56.3 nm±8.0 at L3w period (N = 51, 9 neurites; maximum diameter is 67.5 nm). However, there were no statistically significant differences among the contiguous developmental periods (Fig. 8C). The big dark synaptic vesicles had no statistically significant differences between the first instar larval stage (54.8 nm±8.3, N = 80, 18 neurites) and the second instar larval stage (53.6 nm±7.2, N = 95, 24 neurites). However, the big dark synaptic vesicles did have statistically significant differences between the second instar larval stage and third instar larval stage (55.7 nm±8.7, N = 179, 29 neurites). Moreover, the big dark synaptic vesicles (55.0 nm±8.2, N = 354, 71 neurites) had no statistically significant differences among the first, second, and third instar larval stage (Fig. 8C). In total, the big dark synaptic vesicles in B phase had no statistically significant differences between CV_I_ neurites and CV_II_ neurites (56.3 nm±8.7, N = 68, 14 neurites; maximum diameter 78.8 nm) in the whole instar larval stage (Fig. 8D).

In the larvae stage, the immature T-bar structure in B phase (Fig. 3G–K) was larger than in T phase (Fig. 3E–F, L), and in B phase some of the neurites had large T-bar structures (Fig. 7G). However, there were synapses but no T-bar structure in early stage 17 embryos’ CV_II_ neurites (Fig. 4F).

In the whole first larvae, the big dark synaptic vesicles in CV_I_ neurites was 65.0 nm±17.5 in diameter (N = 72, 21 neurites) in S phase, 60.1 nm±14.3 (N = 86, 24 neurites) in T phase, and 54.8 nm±8.3 (N = 80, 18 neurites) in B phase. Moreover, the big dark synaptic vesicles had no statistically significant differences between S phase and T phase, but had statistically significant differences between S phase and B phase, or T phase and B phase in the first instar larval stage (Fig. 8B).

M phase was the mature stage of the CV neurite development at L3w period, with no microtubules and few big dark synaptic vesicles. However, there were a lot of commensurate synaptic vesicles (32.87 nm±3.93 diameter, n = 277, 8 CV_I_ Neurites; Fig. 8A) distributed around T-bar structure both in CV_I_ neurite (Fig. 3M–N, V; Fig. 5D; Fig. 7I) and CV_II_ neurite (Fig. 4M–N) and there were no synaptic vesicles in center of CV_I_ neurites and CV_II_ neurites. In M phase, some synapses had large T-bar structures (Fig. 3N, O; Fig. 4M–N; Fig. 5D; Fig. 7I), and the mature T-bar structures in CV_I_ neurites (Fig. 3O) were similar to the T-bar structure in NMJ bouton (Fig. 3P) at L3w period. The CV_II_ neurites in M phase remained bifurcated or dendritic (Fig. 4M–N), but most CV_I_ neurites in M phase developed into a globular appearance (Fig. 3M, V; Fig. 5D).

The amount of CV neurites in P, S, T and B phases varied dynamically during larval development. The appearance and length were not different in the first instar larvae (Fig. 1A–C), and the size of the developmental CV neurites is relatively less different (Fig. 1J–K). Therefore we quantified the density and frequency of developmental CV neurites in the first instar larvae (Fig. 8E–G). The density of CV neurites in P phase (Fig. 8E) isat maximum level during L1–2 period (56.5±7.8/mm^2^), and decreased sharply during L1–12 period (9.8±3.1/mm^2^), then remained at a low level during L1–22 period (5.9±2.9/mm^2^) and the following periods (Data not shown). The density of CV neurites in S phase (Fig. 8E) decreased progressively from L1–2 period (416.3±19.5/mm^2^) and L1–12 period (286.1±8.3/mm^2^) to L1–22 period (179.3±12.8/mm^2^). The density of CV neurites in T phase (Fig. 8E) increased significantly from L1–2 period (110.0±7.9/mm^2^) to L1–12 period (172.9±10.8/mm^2^), but decreased from L1–12 period to L1–22 period (134.1±10.3/mm^2^). The density in B phase (Fig. 8E) increased progressively from L1–2 period (69.9±9.6/mm^2^) and L1–12 period (122.1±9.8/mm^2^) to L1–22 period (184.6±8.2/mm^2^). The density of total CV neurites including P, S, T, B phase (Fig. 8G) decreased progressively from L1–2 period (676.0±14.4/mm^2^) and L1–12 period (589.9±19.0/mm^2^) to L1–22 period (505.0±13.0/mm^2^).

The frequency (Fig. 8F) and density of developmental CV neurites were generally consistent in the first instar larvae. The frequency of developmental CV neurites during P phase was the lowest in all the phases, and reduced from L1–2 period (8.4%±0.9) and L1–12 period (1.6%±0.5) to L1–22 period (1.2%±0.6). The frequency of CV neurites in S phase is the highest in all the phases at L1–2 period (62.5%±1.5) and L1–12 period (48.8%±1.7), then reduced progressively from L1–2 period to L1–22 period (35.4%±1.9). The frequency of CV neurites in T phase increased from L1–2 period (16.6%±1.5) to L1–12 period (29.0%±1.1), but decreased at L1–22 period (26.5%±1.5). The frequency of CV neurites in B phase increases progressively from L1–2 period (12.5%±0.6) and L1–12 period (20.5%±1.3) to L1–22 period (37.0%±2.3).

The CV_I_ neurites were produced constantly throughout the larval stage. We could find the P phase at L1–2 period (Fig. 3B), and the P–M phase (Fig. 3Q–V) in L3w period. The distribution of synaptic vesicles was associated with the size of the CV_I_ neurites. Synaptic vesicles were distributed around the large CV_I_ neurites, even in T phase (Fig. 3S) and B phase (Fig. 3J, T–U), but they filled entire small CV_I_ neurites in S phase (Fig. 3D), T phase (Fig. 3 E–F, L) and B phase (Fig. 3G–I, H, K–L).

When the neurites were in M phase, the diameters of CV_I_ neurites (Fig. 3M, V; Fig. 5D; Fig. 7I) and CV_II_ neurites (Fig. 4M–N) both were about 1.5–3 µm.

### 4. Four methods to produce CV_I_ neurites

The CV_I_ neurites were the vast majority of all neurites encountered during the L1–2 (Fig. 1J) to L1–12 periods (Fig. 1K), until the L3w period (Fig. 1L). Our data showed that there were four methods of producing new CV_I_ neurites.

CV_I_ neurites budded at the end of the axons and produced new neurites. We could observe as the neurites budded at the end of axons and the terminal of fine axons was connected to an intumescent neurite (Fig. 5A–E). The developmental processes of the budded neurite included P phase, S phase, T phase (Fig. 5A, C, E), B phase (Fig. 5B–C), and M phase (Fig. 5D) which had the same features as mentioned above. CV_II_ neurites also budded at the end of axon in early stage 17 embryos (Fig. 4B–C).

The neurite budded along the axon and produced a large amount of the new neurites. In early stage 17 embryos, we could observe as a new neurite budded along an axon (Fig. 5E). We could observe three neurites (B phase) budded along an axon at the L1–2 period (Fig. 5G) and at L2–22 period (Fig. 5H). Furthermore, similar phenomenon occurred at the L3–2 period (Fig. 5I). At the L3w period, we observed two axons on both sides of one neurite in T phase (Fig. 5F), which could prove the neurite budded along an axon.

The preexisting neurite budded and developed into one to several new neurites. At first, the preexisting neurite issued several branches outward resembling those in P phase, no microtubules, synapse, or synaptic vesicles (Fig. 6A). Next, the big dark synaptic vesicles joined in (Fig. 6B–C), then the budded branches became bigger (Fig. 6D). We could observe that the preexisting neurite budded into two branches (Fig. 6B–C), or more branches (Fig. 6A, D), with each branch possibly developing a new neurite. Furthermore, we could also observe that the preexisting neurite budded into one branch (Fig. 6E–K), and then divided into two neurites which would go through the S phase (Fig. 6E), T phase (Fig. 6F), B phase (Fig. 6G–K), and M phase (Fig. 6K: left), coupling with the microtubule assembly and polymerization (Fig. 6F–I). In the early stage of the new neurite’s development, the microtubules existed between the two neurites (Fig. 6F–G), the microtubules gradually became shorter (Fig. 6H–I), and finally the microtubules disappeared entirely (Fig. 6J–K).

The bundled axons expanded and formed an irregular neurite. Some main axons gathered into nerve fibers in VNC neuropil region (Fig. 1J–L; Fig. 7A–C), with the diameter of some axons being approximately 0.5–1 µm, and with a similar size to some neurites in B phase (Fig. 7A). Several axons in nerve fibers were enlarged (Fig. 7C–D) and formed a neurite. In the main axon extension process, the axon could form several branches (Fig. 7E–F) and big projections (Fig. 7G–I). The branch, had some big dark synaptic vesicles (Fig. 7E–F), possibly forming a new axon or new neurite, and the projection could form a new irregular neurite which had similar development process and features with CV_I_ neurite. Before the irregular neurite matured, the microtubules and the big dark synaptic vesicles appeared (Fig. 7G). Once the neurite entered in to M phase, the microtubules and big dark synaptic vesicles disappeared and the neurite remained irregular (Fig. 7I).

## Discussion

### 1. Types of Neurites in *Drosophila* VNC Neuropil

According to various criteria, the neurites in *Drosophila* nervous system can be divided into different types. In first instar larval neuropil of *Drosophila* brain and VNC, the terminal neurites are categorized as either globular, varicose, axiform, or dendritiform, according to shape, size, and location [19]. In the *Drosophila* adult brain calyx, there are three morphological and ultrastructural subpopulations of projection neuron boutons according to vesicle phenotype and cytoplasmic electron density: CCV-PNs, DCVPNs and DB-PNs [18]. The *Drosophila* adult NMJs contain two classes of synaptic boutons (type I and II) [14]. However, the *Drosophila* larval NMJs contain three types of synaptic boutons (types I, II, and III) [4], [5] or CV boutons, CV_0_ boutons, DV boutons, and MV boutons [5], according to the features of the synaptic vesicles.

Taking into account that both the NMJs and VNC neurites are issued from the VNC neurons, and the VNC neurites do not have the subsynaptic reticulum (SSR) that are an important basis for the classification of type I, II, and III boutons in the NMJ, so we used the classification criteria described by Jia [5], identifying the four classes of neurites in *Drosophila* VNC neuropil according to their vesicle characteristics: clear vesicle (CV) neurite, dense-core vesicle (DV) neurite, mixed vesicle (MV) neurite, and large vesicle (LV) neurite. The morphological characteristics of the CV neurite, the DV neurite, and the MV neurite in VNC are very similar to the CV bouton, DV bouton, and MV bouton in NMJ. During the L3w period, the CV neurites (Fig. 3A, V; Fig. 4M–N; Fig. 5D; Fig. 7J) in VNC and the CV boutons in NMJ [5] contain small clear vesicles, and the T-bar ultrastructure in the neuropil neurite (Fig. 3O) and NMJ [4], [5] (Fig. 3P) looks very similar. There are some small boutons identified as CV_0_ boutons at Muscles 6, 7, and 13. The CV_0_ boutons contain 44 nm clear vesicles, a few dense-core vesicles of about 94 nm in diameter, and a few large translucent vesicles about 112 nm in diameter. In the L3w period VNC neuropil, no neurites are similar to CV_0_ boutons. The CV neurites were divided into two subtypes, CV_I_ and CV_II_, according to electron density and appearance of the neurite. The CV_I_ neurite is distinguished by light-colored cytoplasm, with most of them being globular at L3w period (Fig. 3A, V; Fig. 5D; Fig. 6J–K). In comparison, CV_II_ neurites have dark cytoplasm, appear bifurcated or dendritic (Fig. 4M–N), and are few in number. There are no NMJ boutons characteristic of CV_II_ neurites. In the adult *Drosophila* mushroom body calyx [6], [23], there are three morphological types of PN boutons (CCV-PNs, DCVPNs and DB-PNs), according to the compositions of synaptic vesicles, with DB-PNs having a dark cytoplasm with both clear and dense-core vesicles [18]. The CV_II_ neurite of the VNC neuropil shares the characteristics of dark cytoplasm of DB-PNs boutons (Fig. 4G–N), but with no dense-core vesicles.

DV boutons of NMJ are filled with spherical dense-core vesicles which can be divided into three discrete populations according to their degree of electron density (dark, intermediate, and light) and size [4], [5]. DV neurites of VNC (Fig. 2B, E, I) are also filled with spherical dense-core vesicles, and can be divided into DV_I_ neurites (Fig. 2E, I) and DV_II_ neurites (Fig. 2B) according to vesicles size. DV boutons have no T-bar structures, based on more than 50 DV neurite observations.

The MV neurites of VNC (Fig. 2C, J) and the MV boutons of NMJ [5] contain both clear and dense-core vesicles. However, in MV neurites, there are several T-bar structures around which both the clear and dense-core vesicles gathered (Fig. 2C), but there are no T-bar structures in MV boutons of NMJ [5]. MV neurites have clear and dense-core vesicles. The clear vesicles were similar in appearance and size (32.2 nm±3.3, N = 69, 7 neurites) to the clear vesicles observed in CV_I_ neurites (Fig. 8A). Furthermore, the dense-core vesicles (64.35 nm±7.4, N = 69, 7 neurites) were similar to dense-core vesicles in DV_I_ neurites in both appearance and size (Fig. 8A), suggesting similar contents in both the MV neurites and DV_I_ neurites. It seems that the MV neurite is more like a mixture of CV_I_ neurites and DV_I_ neurites.

We discovered a previously unreported neurite or bouton in *Drosophila*, which we named the LV neurite. LV neurites were almost filled with spherical translucent and huge vesicles with diameters of 129.5 nm±26.3 (Fig. 2D, F) at L3w period, and have T-bar structures, although number of T-bar structures was very low (Fig. 2F). We observed more than 30 LV neurites, and only one T-bar structure was found during the L3w period of *w^1118^ Drosophila*.

In order to verify the correctness of neurite classification, we observed 10 developmental periods of the *Drosophila*, and the DV neurites, MV neurites, and LV neurites appeared at L1–2 period, but not in early stage 17 embryos.

### 2. *Drosophila* CV neurite morphogenesis

Neurite development is a dynamic process in neuronal migration and differentiation, and it is often packed with synapses, microtubules, and synaptic vesicles, both *in vivo* and *in vitro* [10]. Growth cones are specialized structures at tip of growing neurites or axonal projections. In *Drosophila* embryos, the growth cones reach at the muscle in 13–14 hr AEL [24], [25] with few fluorescence signals of synaptic vesicle marker [24], and there are few synaptic vesicles in the growing neurites [25] in 15 hr AEL. After the immature synapse forms, a few synaptic vesicles cluster in NMJ at late stage 16 embryos [25]. T-bar structures form and a lot of synaptic vesicles cluster from stage 17 embryos [24], [26]–[29] to the larval stage [4], [5], [30] in the NMJ. In the *Drosophila* central nervous system, neurites have different ultrastructures at different developmental stages. The neurite looks like a vacuole [31] at embryonic stage 15, the synapse and the synaptic vesicles form at late stage embryonic/early larval stage [7] and first instar larvae stage [19], [32], and in the adult mushroom bodies [6], [18], [23], the typical T-bar structure forms and a large number of synaptic vesicles fill the neurites.

We observed that the serial developmental processes of the CV neurites, DV neurites, MV neurites, and LV neurites, as well as the morphology of DV neurites (Fig. 2B, E, I), MV neurites (Fig. 2C, J) and LV neurites (Fig. 2D, F, H) did not change significantly. However the morphology of CV neurites underwent dramatic changes (Fig. 3–4; Fig. 9–10). We identified five different developmental phases of CV neurites according characterized by shape, synapse, synaptic vesicles and microtubule: P phase, S phase, T phase, B phase, and M phase.

P phase is the first stage of CV neurites, distinguished by vacuolated projections, in which there were no synapses, synaptic vesicles, microtubules or mitochondria (Fig. 3A–B, Q; Fig. 4A; Fig. 5E;). The growth cone, a dilated terminal of axonal and dendritic processes that acts as a primer when forming the neurite, is absent in cellular organelles, except for microtubules and actin, where it acts as the sensory and motile organelle of developing neurons [33]. Therefore, the neurite in P phase is very similar to a growth cone in that the neurite has microtubules in S phase, it appears that the neurite in P phase forms earlier than growth cones. There are significant morphological differences between the central nervous system (VNC) and the peripheral nervous system (NMJ). The growth cones reach the muscle in NMJ at 13–14 hr AEL, they are flat and dendritic, and there are several long filopodia [24] and lamellipodia [33]. In contrast to VNC neurite, the P neurite is spherical or globular, with no filopodia or lamellipodia around it. Since neurites in P phase are rarely encountered in the larval stage (Fig. 3Q; Fig. 8E–F), this suggests that P neurites develop quickly.

The next two phases of neurite development we identified were S phase and T phase. The neurite is transported some mitochondria (Fig. 3C–D, R–T; Fig. 4B; Fig. 7D), as well as a few different synaptic vesicles (Fig. 3C–D; Fig. 4B) or microtubules (Fig. 3C; Fig. 4B) in S phase. In T phase, neurites have immature synapses. There are small T-bar structures in T phase of CV_I_ neurites, while CV_II_ neurites have electron-dense synapses without T-bar structures during T phase in early stage 17 embryos.

In B phase, the appearance of globular neurites is remodeled, and they subsequently appear bifurcated or dendritic. Most of the T-bar structures were incomplete and consequently appeared either too small (Fig. 3G–K), or contained gap in the T-bar structure (Fig. 4G–H) at the larval stage in both CV_I_ and CV_II_ neurites. The two different diameters of synaptic vesicles were present and filled throughout B phase (Fig. 3G–K; Fig. 4G–L). The synapse did not contain T-bar structures during B phase in CV_II_ neurites (Fig. 4F).

In M phase, the structures of the CV neurite in the VNC and the CV bouton in the NMJ [4], [5] are very similar (Fig. 3O–P). The neurites (both CV_I_ and CV_II_) have a lot of small commensurate synaptic vesicles that cluster around the T-bar structure. The shape of CV_II_ neurites remained bifurcated or dendritic in M phase (Fig. 4M–N), but most of the CV_I_ neurites became globular again (Fig. 3M, V; Fig. 5D). If the big dark synaptic vesicles are present in the neurite, even the typical T-bar structure can be observed, then the neurite is in B phase (Fig. 7G), not in M phase.

During the CV neurite development of *Drosophila* VNC, microtubules, mitochondria, synapses, and synaptic vesicles are the four elements visible using TEM. The neurite appears bubbly in P phase, and the organelles, such as mitochondria, microtubules, and a few synaptic vesicles, are transported in the neurite during S phase. The immature synapses form at T phase, coupled with the arrival of more synaptic vesicles. Taking into account that the neurites at P phase, S phase, and T phase are globular, and that they develops fast, we conclude that P phase, S phase, and T phase are the early phases of CV neurite development. In B phase, the shape of globular neurite is reorganized, and we conclude that B phase is the transitional phase of CV neurite development. In M phase, the neurites have an abundance of small commensurate synaptic vesicles that cluster around the T-bar structure, and the structural features are very similar to the bouton of third instar larval NMJ, therefore M phase is the mature phase of CV neurite development. The big vesicles are clear in the endocytosis disorder of some NMJ mutations, but the big vesicles are dark in S phase, T phase, and B phase of neurite. The big dark vesicles appear in S phase, T phase, and B phase, but almost disappear in M phase. The big dark synaptic vesicles have no statistically significant differences among the contiguous developmental periods (Fig. 8C). However, they do have statistically significant differences between the second instar larval stage and third instar larval stage. Moreover, the big dark synaptic vesicles in the whole larval stage showed no statistically significant differences among the first instar larval stage, the second instar larval stage, and third instar larval stage (Fig. 8C). In total, the big dark synaptic vesicles in B phase have no statistically significant differences between CV_I_ neurites and CV_II_ neurites throughout the entire instar larval stage (Fig. 8D). Thus, we speculate the big dark vesicles have some function during the maturation of the CV neurite.

Most CV_I_ neurites have a diameter of about 1.5–3 µm (Fig. 3M, V; Fig. 5D), and they have the largest number of all the axonal neurites (Fig. 1J–L). This means that the CV_I_ neurites should be suitable to analyze their morphology and display specific subcellular localization of proteins by light microscopy, and they should serve as a potential model in CNS *in vivo*.

### 3. The manners to produce CV_I_ neurites

As *Drosophila* develop from the first instar to third instar, the number of NMJ boutons increase 10-fold [4], [21]. The new NMJ boutons form and increase by the “bud”, “divide”, or “de novo” mechanisms [22].

In CNS of vertebrates and invertebrates, most neurites bud along the long axon *in vivo* [22], [34], [35] or *in vitro*. Once new neurites or growth cones bud along the long axon, they will develop into a large amount of the mature neurites in *Drosophila* VNC (Fig. 5G–I; Fig. 9) and NMJ [22]. Growth cones are the sensory and motile organelles of developing neurons [33], and paly a critical role in forming new neurites. Taking into account that the axons are very long and that the neurites have a bead-like distribution (Fig. 5G–I; Fig. 9) along axons [34], and that there are a significant number of neurites in S phase and T phase during the L1–2 (Fig. 1J) and L1–12 periods (Fig. 1K), we conclude that it is the primary method of forming new neurites along the long axon in *Drosophila* VNC (Fig. 9).

During CV neurite development in *Drosophila* VNC, there is a special B phase when the structure of neurite is reconfigured. The shape changes, one or several branches bud from the neurite (Fig. 6; Fig. 9), which in turn produces one or several new neurites. This mechanism of budding is an important method of forming new neurites from the pre-neurite [22]. Some irregular neurites form in main axons, containing mature T-bar structures and many synaptic vesicles, which may be a special mechanism of increasing the number of synapses (Fig. 7I) and the efficiency of transferring nerve signals, such as multiple postsynaptic elements [6]–[9]. Before completely developing into M phase, the neurites contain microtubules, big dark vesicles, or immature T-bar structures. In M phase, there are big dark vesicles and some typical T-bar structures in the neurites, but no microtubulues (Fig. 3M, V; Fig. 4M–N; Fig. 5D; Fig. 7I), which means the microtubules, big dark vesicles, and the immature T-bar structure are the markers that the neurite is developing.

### 4. The model of CV neurite development

According to our experimental results, we propose the following model of CV neurite development. In CV_I_ neurites during P phase, the neurite buds at the end of the axon (Fig. 9A), where there are few cell contents, as described in Fig. 3A–B, Q, and reported by Jacobs and Goodman [31]. Then some cell contents, such as mitochondria, synaptic vesicles, or microtubules, are transported into the neurite, and the P phase enters into S phase (Fig. 9B; Fig. 3C–D, R; Fig. 6E; Fig. 7C–D). With axon extension, some new neurites in P phase bubble along the axon (Fig. 9C; Fig. 5E; [34]). At the same time, the new neurite that is budded at the end of the axon from S phase enters into T phase (Fig. 9C; Fig. 5A, C, E; [34]). Next, the neurites that budded along the axon develop into T phase (Fig. 9D; Fig. 5F), and the neurites distribute like beads along the axon (Fig. 9D; [34]). Next, some neurites in T phase enter into B phase, and new neurites bud from the preexisting neurites (Fig. 9E; Fig. 6A–C). Next, the neurites that budded from preexisting neurites enter S phase (Fig. 9F; Fig. 6E). At last, all the neurites respectively enter into T phase, B phase, or M phase. During M phase, most of the neurites are globular and have intensive commensurate synaptic vesicles clustered around the typical T-bar structures, few big dark synaptic vesicles are found in the neurite, and no synaptic vesicles are in the center of the neurite (Fig. 9G; Fig. 3M, V; Fig. 4M–N; Fig. 5D; Fig. 7I).

In CV_II_ neurites, the early phases are similar to CV_I_ neurites. The CV_II_ neurite in the P phase (Fig. 10A; Fig. 4A), the S phase (Fig. 10B; Fig. 4B), the T phase (Fig. 10C; Fig. 4C–D), the B phase (Fig. 10D–E; Fig. 4B; and Fig. 4E–L). Once CV_II_ neurites enter M phase, they have dark cytoplasm, appear bifurcated or dendritic, and possess intensive commensurate synaptic vesicles clustered around the typical T-bar structure (Fig. 10F; Fig. 4M–N).

### 5. Dominant factors of *Drosophila* VNC neuropil growth

In general, VNC is developed in low evolutionary invertebrates. During *Drosophila* development, the proportion of VNCs is gradually reduced in the CNS (Fig. 1A–I), and the volume of VNC is still much greater than that of the brain until the late third instar larvae (Fig. 1I), and even accounts for a considerable proportion of the nerve tissue in the adult *Drosophila* [18].

The development of *Drosophila* larval VNC is a non-linear process. VNC developed slowly in *Drosophila* first instar larvae (Fig. 1A–C), and developed fast in *Drosophila* second instar larvae (Fig. 1D–F) and third instar larvae (Fig. 1G–I). The area (Fig. 1J–L) of cross-section and length (Fig. 1A–I) of the VNC neuropil significantly increased from *Drosophila* first instar larvae to third instar larvae. All the neuropils are wrapped by inner glial cells [36]–[40], as shown by the white triangle (Fig. 1J–L). The CV_I_ neurites, occupying most of the VNC neuropil, have the greatest contribution to the growth of VNC neuropil. The volume of CV_I_ neurites gradually increase from P phase to M phase (Fig. 3; Fig. 9), and the number of CV_I_ neurites gradually increase in four manners (Fig. 5–7; Fig. 9), and thus these two factors induce growth of *Drosophila* VNC neuropil.

## Materials and Methods

### Fly stocks and defining the developmental periods

The wile-type flies were *w^1118^*. All the flies were raised on the standard cornmeal-dextrose agar media at 25°C under a 12 hr light: 12 hr dark (LD) cycle. For the embryonic analyses, the flies of 6–9 days post-eclosion were gathered on the yeasted apple juice agar plates in dark, 20 minutes later, the eggs were collected, and the embryonic stages were given according to Campos-Ortega and Hartenstein [41]. To identify the larval developmental periods, the eggs on the plates were transferred to the glasses tubes filled with standard fly food at a density of 20 individuals per a glass tube. The larvae were divided into nine specific periods. Briefly, the time point when the early 1st instar developed 2 hr was named L1–2 period, the time point when the middle 1st instar developed 12 hr was named L1–12 period, and the time point when the late 1st instar developed 22 hr was named L1–22 period. The names of the L2–2 period, L2–12 period, L2–22 period, L3–2 period, and L3–12 period were named in the same manner, and the late 3rd instar wandering was named the L3w period.

### Electron microscopy procedure in embryos and larvae

Embryos were prepared for TEM using standard techniques [7], [29]. Briefly, the early stage 17 embryos (17 hr after egg laying at 25°C) were manually dechorionated and injected with fixative (5% glutaraldehyde in 0.1 M sodium cacodylate buffer, PH 7.4). The embryos were transferred to 2.5% glutaraldehyde in 0.1 M sodium cacodylate buffer (PH 7.4) for 30 min, cut embryos ends with a sharp blade, and then transferred to 2.5% glutaraldehyde in 0.1 M sodium cacodylate buffer over night at 4°C. The brains and VNCs of the larvae were dissected in Jan solution (128 mM NaCI, 2 mM KCl, 4 mM MgCI, 35 mM sucrose, 5 mM Hepes, PH 7.4) within 20 minutes, and fixed in a mixture of 2% glutaraldehyde and 2% formaldehyde in 0.1 M sodium cacodylate buffer (PH 7.4) at 4°C overnight. All the specimens were washed in 0.1 M sodium cacodylate (PH 7.4) buffer containing 264 mM sucrose four times, transferred to 1% osmium tetroxide in 0.1 M sodium cacodylate (PH 7.4) for 2 hr, washed three times in ddH_2_O, and stained in 2% aqueous uranyl acetate for 2 hr. The specimens were dehydrated in an ethanol series, passed through propylene oxide two times, and embedded into a sheet about 1–2 mm in Epon812 (SPI Science) to allow the image of VNC to be captured with the Olympus microscope. Ultra-thin sections (90 nm thick) that were cut from the VNC cross-section of the abdominal segments 2–4 with Leica UC6 using a diamond knife. Grids were post-stained with 2% saturated uranyl acetate in 50% ethanol and 1% lead citrate (PH 12), examined with H-7650 electron microscope and recorded with a Ganton 830 digital CCD. More than 3 animals for each developmental period were analyzed. The electron microscope procedure of NMJ sample: See accompanying article by Atwood et al. [4].

### Statistical analysis

The synaptic vesicles for measuring the diameter of CV neurite, DV neurite, MV neurite, and LV neurite were collected from L3w period. The big dark synaptic vesicles in S phase and T phase for measuring the diameter of the CV_I_ neurite were collected from L1–2 period, L1–12 period, and L1–22 period. The big dark synaptic vesicles in B phase were collected throughout the larval stages. The counted clear vesicles were less than 45 nm in L3w period, and the counted big dark vesicles were more than 45 nm in S phase, T phase and B phase. For statistic analysis of CV neurites, the images were collected by electron microscope at 12000× magnification in the first instar larvae. The data were measured using Image J (NIH), and the data were analysized using GraphPad Prism.
